# Ontogenesis of peptidergic neurons within the genoarchitectonic map of the mouse hypothalamus

**DOI:** 10.3389/fnana.2014.00162

**Published:** 2015-01-12

**Authors:** Carmen Díaz, Nicanor Morales-Delgado, Luis Puelles

**Affiliations:** ^1^Department of Medical Sciences, School of Medicine and Institute for Research in Neurological Disabilities, University of Castilla-La ManchaAlbacete, Spain; ^2^Department of Human Anatomy and Psychobiology, University of Murcia, School of Medicine and IMIB (Instituto Murciano de Investigación Biosanitaria)Murcia, Spain

**Keywords:** hypothalamus, neuropeptides, genoarchitecture, progenitor areas, migrations

## Abstract

During early development, the hypothalamic primordium undergoes anteroposterior and dorsoventral regionalization into diverse progenitor domains, each characterized by a differential gene expression code. The types of neurons produced selectively in each of these distinct progenitor domains are still poorly understood. Recent analysis of the ontogeny of peptidergic neuronal populations expressing *Sst, Ghrh, Crh* and *Trh* mRNAs in the mouse hypothalamus showed that these cell types originate from particular dorsoventral domains, characterized by specific combinations of gene markers. Such analysis implies that the differentiation of diverse peptidergic cell populations depends on the molecular environment where they are born. Moreover, a number of these peptidergic neurons were observed to migrate radially and/or tangentially, invading different adult locations, often intermingled with other cell types. This suggests that a developmental approach is absolutely necessary for the understanding of their adult distribution. In this essay, we examine comparatively the ontogenetic hypothalamic topography of twelve additional peptidergic populations documented in the *Allen Developmental Mouse Brain Atlas*, and discuss shared *vs*. variant aspects in their apparent origins, migrations and final distribution, in the context of the respective genoarchitectonic backgrounds. This analysis should aid ulterior attempts to explain causally the development of neuronal diversity in the hypothalamus, and contribute to our understanding of its topographic complexity in the adult.

## Introduction

The hypothalamus is a complex forebrain structure that regulates vital processes and various visceral and somatic behavior. Numerous hypothalamic peptides are involved in modulating such functions. Advances on the molecular mechanisms associated to the ontogeny of peptidergic neurons are helping us to understand developmental defects that cause metabolic and neuroendocrine disorders (Michaud, 2001; Caqueret et al., 2005). Murine experimental studies in which some transcription factors were inactivated, showed that gene products such as GSH1, MASH1, SIM1, SIM2, ARNT2, BRN-2, and OTP are crucial for the differentiation of the parvicellular GHRH, SST, TRH, CRH neuroendocrine cell types, as well as the magnocellular OT and VP secreting neurons (Nakai et al., 1995; Schonemann et al., 1995; Li et al., 1996; Michaud et al., 1998; Acampora et al., 1999; Wang and Lufkin, 2000; Hosoya et al., 2001; Goshu et al., 2004; Caqueret et al., 2005; McNay et al., 2006; Szarek et al., 2010).

Furthermore, developmental gene expression patterns allowed a molecular characterization of specific areal subdivisions within the alar and basal regions of the hypothalamus. Such regional analysis produces *genoarchitectonic maps* (Ferran et al., 2009; Puelles and Ferran, 2012), which illuminate the variety of molecular mechanisms controlling the specification of differential neuronal fates at each particular hypothalamic subregion. Each neuroepithelial area of the hypothalamus becomes distinct by its unique gene expression profile. As a progenitor domain, it is capable of controlling differentially over time its proliferative and neurogenetic activity, producing specific neuronal populations, and simultaneously generating signals for axonal and neuronal navigation. Different neuron types may originate sequentially at the same domain, due to temporal changes in the local molecular profile. Some neurons migrate tangentially within the mantle layer, invading other hypothalamic domains before they finish developing their phenotype and acquire functional roles within particular circuits.

Tangential migrations from nearby forebrain areas into the hypothalamus, as well as internal migrations, are not exceptional during hypothalamic development (e.g., Keyser, 1972; Alvarez-Bolado et al., 2000; Skidmore et al., 2008; Zhao et al., 2008). Puelles et al. (2012) postulated several other tangential migrations, including distinct cell streams forming the ventromedial nucleus and the ventral premamillary nucleus. Our recent studies likewise suggested widespread tangential migrations of some peptidergic neurons (Morales-Delgado et al., 2011, 2014). As a result, a frequent feature of conventional hypothalamic nuclei is their content of mixed neuron types, using a variety of neuropeptides and neurotransmitters; this intermixing may be functionally relevant (Rhodes et al., 1981; Sofroniew and Glasmann, 1981; Markakis and Swanson, 1997; Broberger et al., 1998; Elias et al., 1998; Sawchenko, 1998; Broberger, 1999; Simmons and Swanson, 2009; Shimogori et al., 2010; Puelles et al., 2012; Tobet and McClellan, 2013).

**Figure 1:**
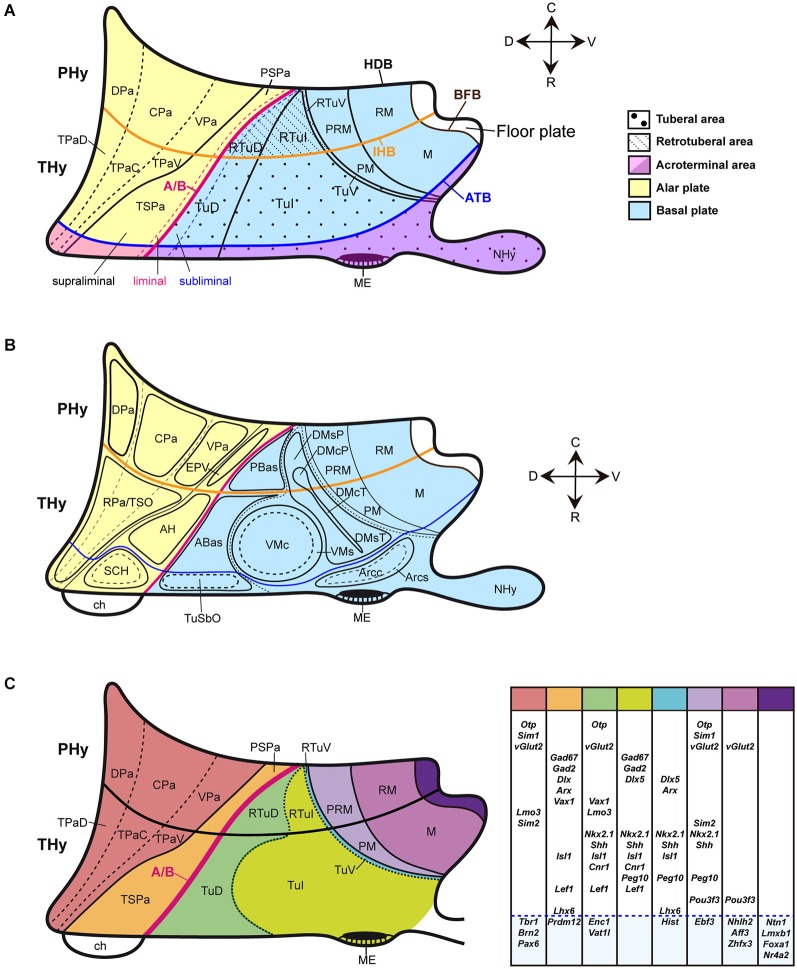
**Schematic partial prosomeric model of the forebrain representing the general position, morphologic organization and principal nuclear and genoarchitectonic subdivisions of the hypothalamus**. The rostrocaudal (R-C) and dorsoventral (D-V) spatial directions are indicated. **(A)** Schema of the main hypothalamic progenitor areas distributed across the dorsoventral and rostrocaudal dimensions. The longitudinal alar/basal boundary (A/B), and the intrahypothalamic (IHB) and acroterminal (ATB) boundaries are indicated respectively as thick pink, orange and blue lines. The hypothalamic area is subdivided rostrocaudally into neuromeric peduncular and terminal parts (PHy, THy). Alar territories are shown on the *left* (yellow) and basal territories on the *right* (blue). The alar hypothalamus is subdivided into the paraventricular (TPa/PPa) and subparaventricular (TSPa/PSPa) areas (each pair of areas refers to THy and PHy components of a longitudinal zone), plus corresponding acroterminal subregions. The paraventricular area shows a general tripartition into dorsal, central and ventral parts (TPaD, TPaC, TPaV, DPa, CPa, VPa). The subparaventricular area appears subdivided into *supraliminal* and *liminal* parts (referring to the alar-basal limit; the liminal domain expresses *Nkx2.2*). The basal hypothalamus is also divided dorsoventrally into the large tuberal/retrotuberal (Tu/RTu) area and the primary mamillary/retromamillary (M/RM) area, plus the corresponding acroterminal subregions. The THy/PHy parts of the hypothalamic floor lie underneath (white). Moreover, the Tu/RTu region is subdivided into three dorsoventral parts: TuD/RTuD, TuI/RTuI and TuV/RTuV. The TuD/RTuD contains a dorsal *subliminal* part (which also expresses *Nkx2.2*). The primary M/RM area is subdivided into a perimamillary/periretromamillary band (PM/PRM), and the underlying secondary M/RM complex proper. **(B)** Map of the main alar and basal hypothalamic nuclei represented upon the diagram shown in **(C). (C)** Schematic color-coded map of the gene expression patterns distributed across the main dorsoventral hypothalamic subdivisions of E13.5 mouse embryos. Varied combinations of gene markers create a characteristic molecular profile for each territory, as represented in tabular form on the *right*. Genes expressed in more than one dorsoventral domain are grouped in the white area and genes expressed in an unique domain are shown in the blue area. For abbreviations, see the list.

Puelles et al. (LP, online reference atlases and ontology of the *Allen Developing Mouse Brain Atlas*, 2009; Puelles et al., 2012, 2013) recently updated the prosomeric model, particularly as regards the hypothalamus. It was defined as a bi-neuromeric rostral forebrain territory, lying in front of the diencephalon proper and ventral to the telencephalon (which can be seen as a part of it; Figure 1A). The *Mash1/Dlx/Arx/Isl1*-expressing preoptic area was ascribed to the subpallial telencephalon. The neighboring *dorsal* part of the alar hypothalamus—the paraventricular area- singularly expresses the genes* Otp* and* Sim1*. There is thus a clearcut molecular hypothalamo-telencephalic boundary, which is longitudinal (Shimogori et al., 2010; Puelles et al., 2012). At its *ventral* end, the hypothalamus is represented by the mamillary and retromamillary areas, which include at their median plane the *rostral end* of the forebrain floor plate. The hypothalamus has no rostral neighbor, since it represents the rostralmost part of the neural tube. As a consequence, the right and left lateral walls of the neural tube are here continuous one with another *primarily* (from neural plate stages onwards), creating an unique *median* alar + basal subregion recently named “acroterminal area” (Puelles et al., 2012). Terminal lamina, optic chiasma and tuberomamillary midline specializations (e.g., median eminence and neurohypophysis) develop there. The roof plate is telencephalic (partly choroidal and partly septocommissural), and ends at the anterior commissure.

The Figures 1A–C illustrate the hypothalamic progenitor domains defined within the prosomeric model, with correlative major derived nuclei, and basic genoarchitectonic patterns. Additional such data will be provided below, as each domain is considered. There is a rostrocaudal partition of the hypothalamus and attached telencephalon into two transverse (neuromeric) parts (hypothalamic prosomeres 1 and 2, or hp1/hp2; Pombal et al., 2009; Nieuwenhuys, 2009; Puelles et al., 2012). To avoid confusion with older terminologies, the resulting two transverse parts of the hypothalamus were renamed as *terminal* and *peduncular* hypothalamus (THy, PHy), referring to the terminal rostral position of the former and to the association of the latter with the course of peduncular telencephalic fibers (Puelles et al., 2012; for genoarchitectonic analysis of these rostrocaudal hypothalamic divisions, see Ferran et al., under review).

THy and PHy are both divided dorsoventrally into longitudinal alar, basal and floor territories, which display differential molecular profiles and various microzonal subdivisions. These longitudinal zones are continuous *caudally* with diencephalic counterparts, being a result of shared dorsoventral patterning. The alar hypothalamus is divided into the *paraventricular area* (continuous with the prethalamic eminence and the prethalamic reticular nucleus), and the *subparaventricular area* (continuous with the prethalamic zona incerta) (TPa, PPa; TSPa, PSPa; Figures 1A–C; Puelles et al., 2012). The narrow TPa contains the eye vesicle, the optic stalk and the chiasma. The SPa contains subpially the optic tract.

The basal hypothalamus largely corresponds to the classical *tuberal* and *mamillary* hypothalamic regions. However, these two regions belong exclusively to the voluminous basal THy. The new terms *retrotuberal* and *retromamillary* regions are needed for the corresponding PHy basal territories; accordingly, the tuberal and retrotuberal domains compose a hypothalamic longitudinal column, and the mamillary and retromamillary domains another (Tu/RTu; M/RM; Figures 1A–C; note “retromamillary” substitutes for the older “supramamillary” term). Tu/RTu further subdivides dorsoventrally into dorsal, intermediate and ventral longitudinal microzonal domains (TuD/RTuD, TuI/RTuI, TuV/RTuV; Figures 1A–C). The TuD/RTuD domain corresponds to the classic, precociously differentiated “hypothalamic cell cord’ (Gilbert, 1935; Keyser, 1972). The TuI/RTuI contains the ventromedial, dorsomedial and arcuate nuclei, whereas the narrow TuV/RTuV produces the hypothalamic histaminergic neurons (Puelles et al., 2012). The underlying mamillary/retromamillary basal territory also shows a molecularly distinct dorsal microzonal subdivision, the perimamillary/periretromamillary domain (PM, PRM; Figures 1A–C; Bardet et al., 2008; Puelles et al., 2012; Morales-Delgado et al., 2014; Allen Developing Mouse Brain Atlas).

**Figure 2:**
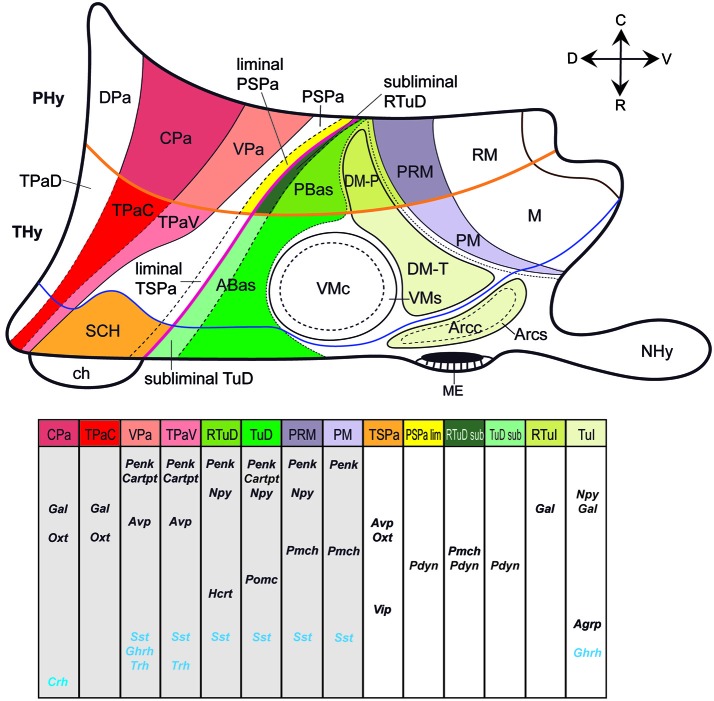
**Schematic color-coded prosomeric map illustrating the main prenatal and early postnatal progenitor areas where peptidergic cell populations originate in the mouse hypothalamus**. Specific peptidergic transcripts generated at each source area are represented in tabular form; each column corresponds to a progenitor area identified by a color in the upper schema. For abbreviations, see the list.

This anteroposterior and dorsoventral map of the hypothalamus provides a scenario where the genoarchitectonically characterized progenitor domains of specific peptidergic neuron groups can be precisely identified (Figures 1C, 2). Moreover, jointly with results from knockout and other transgenic mice, this modern hypothalamic scenario potentiates the analysis of patterning mechanisms implicated in neuronal type specification.

## Material

**Figure 3:**
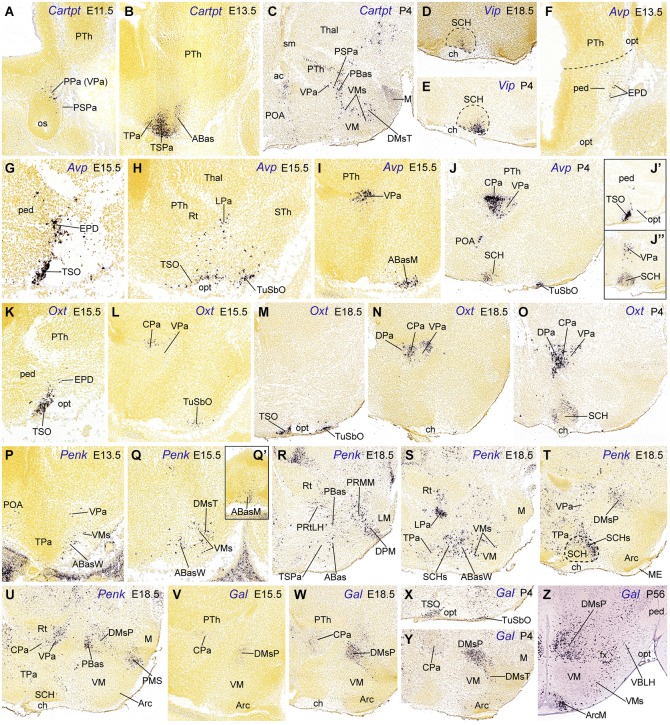
**Sagittal sections through the mouse hypothalamus at embryonic and postnatal days (A–Y), plus a transverse section at an adult stage (P56, Z), showing relevant examples of the hypothalamic expression of *Cartpt, Vip, Avp, Oxt, Penk* and *Gal* mRNas. (A–C)** *Cartpt*. **(D,E)** *Vip*. **(F–J”)** *Avp*. **(K–O)** *Oxt*. **(P–U)** *Penk*. **(V–Z)** *Gal*. All images were downloaded from the Allen Developing Mouse Brain Atlas.^2^ For abbreviations, see the list.

**Figure 4:**
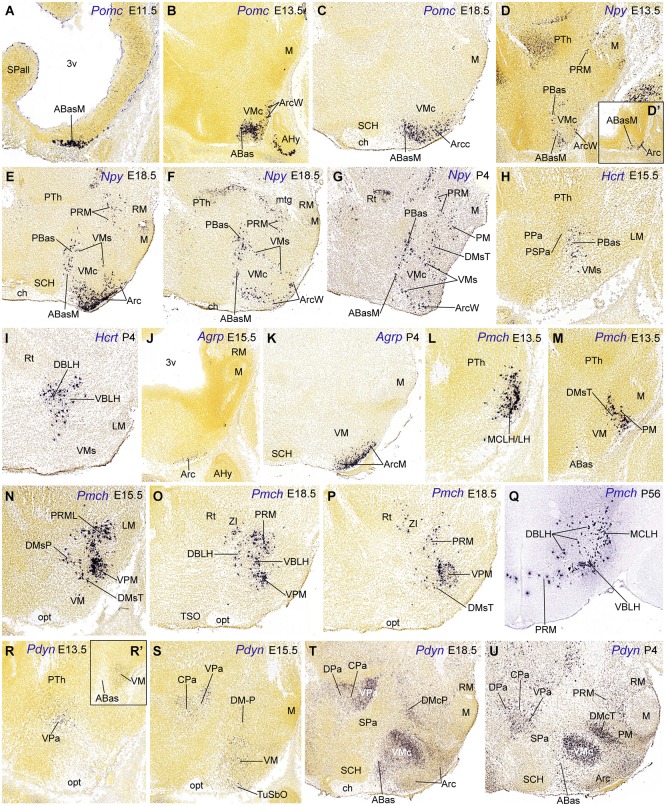
**Parasagittal (A–P, D’, R–U, R’) and transverse (Q) sections through the hypothalamus at embryonic and postnatal stages, showing relevant examples of the spatiotemporal expression of *Pomc* (A–C), *Npy* (D–G), *Hcrt* (H–I), *Agrp* (J–K), *Pmch* (M–Q) and *Pdyn* (R–U)**. All images were downloaded from the Allen Developing Mouse Brain Atlas.^3^ For abbreviations, see the list.

**Figure 5:**
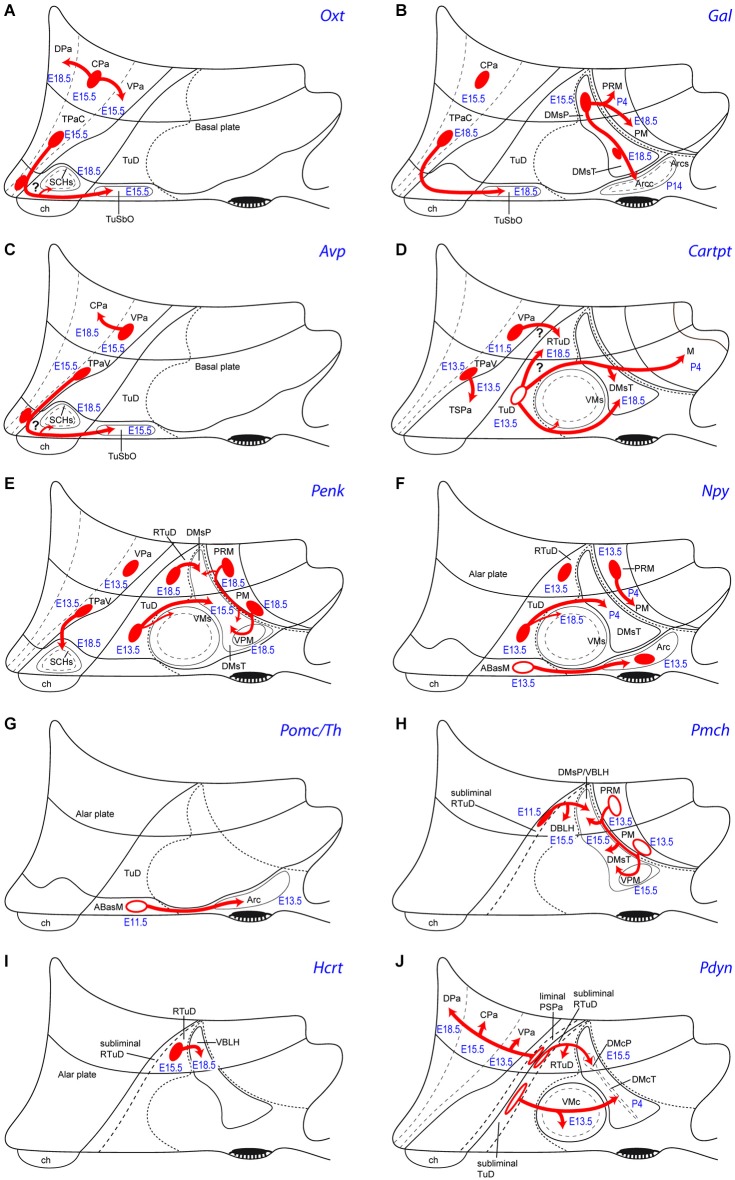
**Schemata of the apparent tangential migration routes (red arrows) of the *Otx (A), Gal (B), Avp (C), Cartpt (D), Penk (E), Npy (F), Pomc/Th (G), Pmch (H), Hcrt (I) and Pdyn (J)* cell populations, highlighting the source areas (red ovals) and the apparent recipient areas (named areas next to arrow tips)**. Uncertain postulated migrations are indicated with a question mark. Filled red ovals identify progenitor areas where the relevant peptidergic cells persist and accumulate radially (see manuscript for details). Empty red ovals mark instead the progenitor areas whose mapped peptidergic population entirely moves tangentially into the indicated recipient areas (devoid of the cell type in the adult). The schemata also show the recorded embryonic/postnatal stages of first appearance of a particular peptidergic population at its source, as well as at individual recipient areas. For abbreviations, see the list.

We examined the development of twelve peptidergic cell populations (*Agrp, Avp, Cartpt, Gal, Hcrt, Npy, Oxt, Penk, Pdyn, Pmch, Pomc, Vip*; Table 1), analyzed from *in situ* hybridization images downloaded from the *Allen Developing Mouse Brain Atlas*.^1^ These are mostly sagittal sections; while this section plane is appropriate for the analysis of potential dorsoventral and anteroposterior tangential migrations, the visualization of some anatomic landmarks may be compromised. We recurred to careful analysis of all sagittal (eventually also coronal) section planes shown at the *Allen Atlas*, as well as to our extensive experience with multiple planes of sections through the mouse hypothalamus. We correlated the positions of peptidergic cells at the time points available at the *Allen Atlas* (embryonic days E11.5, E13.5, E15.5 and E18.5, and postnatal day P4) with the genoarchitectonically distinct areas (Figure 1C) and conventional nuclei (Figure 1B), following the model of Puelles et al. (2012). We also recorded aspects of areal heterochrony (Table 2), and apparent tangential migrations (Table 3; Figures 3–5). Note that any interpretations of “migration” extracted from our descriptive material are necessarily hypothetic, open to experimental testing, though we hold that these conclusions momentarily represent the most parsimonious interpretations of the data. For overview, we added the similar data on *Sst, Ghrh, Trh* and *Crh* neurons of Morales-Delgado et al. (2011, 2014) in our present Figure 2; Tables 2, 3. Our discussion accordingly contemplates sixteen peptidergic cell types, allowing some general conclusions.

**Table 1:** **Peptide mRNAs mapped in this work**.

*Agouti-related peptide (Agrp)*
*Arginin-vasopressin (Avp)*
*Cocaine- and amphetamine-regulated transcript prepropeptide (Cartpt)*
*Galanin (Gal)*
*Hypocretin/orexin (Hcrt)*
*Neuropeptide Y (Npy)*
*Oxytocin (Oxt)*
*Preproenkephalin (Penk)*
*Prodynorphin (Pdyn)*
*Pro-melanin-concentrating hormone (Pmch)*
*Pro-opiomelanocortin α (Pomc)*
*Vasoactive intestinal polypeptide (Vip)*

*All transcript patterns correspond to the Allen Developing Brain Mouse Atlas database*.

**Table 2:** **Timing of earliest differentiation of the studied peptidergic cell types within particular hypothalamic progenitor areas**.

Terminal areas		E10.5	E11.5	E12.5	E13.5	E15.5	E.18.5
**TPa**	TPaD
	TPaC					*Oxt*	*Gal*
	TPaV			***Trh*** ***Ghrh***	*Cartp*; ***Sst***	*Avp*	*Penk*
**TSPa**	TSPa				*Cartpt*		*Vip* *Avp?* *Oxt?* (SCH)
**Tu**	TuD (ABas)	***Sst***	*Pomc*		*Cartpt* *Npy* *Penk*
	TuI				***Ghrh*** (Arc) *Npy* (Arc) *Pdyn* (VM)	*Agrp* (Arc)	*Gal* (DM-T)
	TuV
**PM**	PM				*Pmch*		*Penk* *Gal* ***Sst***
**M**	M
**Peduncular areas**		**E10.5**	**E11.5**	**E12.5**	**E13.5**	**E15.5**	**E.18.5**
**PPa**	DPa
	CPa				***Crh***	*Oxt* *Gal*
	VPa	***Trh*** ***Ghrh***	*Cartpt*	***Sst***	*Avp* *Penk* *Pdyn*
**PSPa**
**RTu**	RTuD (PBas)		*Pmch*		*Penk* *Npy* *Sst*	*Hcrt*
	RTuI (DM-P)					*Gal* *Pdyn*
	RTuV
**PRM**	PRM				*Npy* *Pmch* *Sst*		*Penk*
**RM**	RM

*Light gray areas are devoid of peptidergic cells, whereas dark gray areas produce two or more types simultaneously. Orange background: Alar domains. Reddish background: Basal domains. Bold letters: peptidergic cells studied in Morales-Delgado et al., 2011; Morales-Delgado et al., 2014; Morales-Delgado, 2012. All other peptidergic cell types were analyzed from Allen Developing Mouse Brain Atlas datasets*.

**Table 3:** **Alar/basal topography of the different peptidergic cell populations, according to their primary progenitor sources and their perinatal distribution, after tangential migration**.

Progenitor sources			Perinatal distribution		
Alar	Alar+Basal	Basal	Alar	Alar+Basal	Basal
*Oxt*	*Gal*	*Agrp*	*Vip*	Oxt*, ***Oxt***	*Agrp*
*Avp*	*Cartpt*	*Npy*	Crh*	Avp*, ***Avp***	Npy*
*Vip*	*Penk*	*Pomc*		Gal*	Pomc*
*Crh*	*Pdyn*	*Pmch*		Cartpt*	Pmch*
*Trh*	*Sst*	*Hcrt*		Penk*	Hcrt*
	*Ghrh*			Pdyn*	Ghrh*
				Sst*	***Ghrh***
				Trh*, ***Trh***

*Bold tags: populations that move from alar into basal domains. Asterisks: populations that move within their primary domain*.

## Results and discussion

Figure 2 maps the locations where the different peptide markers listed in Table 1 first appear expressed, adding the results of Morales-Delgado et al. (2011, 2014) and Morales-Delgado (2012). It reveals that given dorsoventrally disposed longitudinal zones dominate as major sources of neuropeptidic populations. These are the Pa area in the alar hypothalamus and the TuD/RTuD and PM/PRM areas in the basal hypothalamus. It is also apparent that multiple peptidergic cell types emerge within these domains, and, remarkably, some cell types appear independently in two or all three of them. A shared characteristic of the molecular profiles of these three areas is the expression of the transcription factor *Otp* (Figure 1C; Morales-Delgado et al., 2011, 2014; Morales-Delgado, 2012; Puelles et al., 2012). This was already shown to be required for the differentiation of a number of peptidergic cell types (Acampora et al., 1999; Wang and Lufkin, 2000). A few other progenitor domains, such as SCH within the acroterminal part of alar TSPa, and DM-P, DM-T, and Arc within the basal hypothalamus, give rise to other peptidergic derivatives, in a more restricted mode. On the other hand, there are hypothalamic progenitor domains that so far do not represent sources of peptidergic neurons. These results are also represented in tabular form within Figure 2 and in Table 2.

### Alar hypothalamic sources: the paraventricular area

This area consists of a large, triangular peduncular paraventricular subarea (PPa) defined by diverse deep populations of the *main paraventricular nucleus* (Pa), and a thin terminal paraventricular subarea (TPa), which develops the deep *rostral paraventricular nucleus* (RPa; the conventional “anterior paraventricular area”, or aPV), and the subpial *supraoptic nucleus* (PPa; TPa; RPa; TSO; Figure 1A). The main Pa is subdivided into dorsal, central, and ventral parts (DPa, CPa, VPa; Figure 1B). Puelles et al. (2012) showed that the PPa/TPa essentially contains glutamatergic cells.

The paraventricular area originates peptidergic cell types expressing *Gal, Penk, Cartpt, Avp* and *Oxt* (present data) as well as *Sst, Trh, Ghrh*, or *Crh* cells (Figure 2; Jing et al., 1998; Morales-Delgado et al., 2011, 2014), with timing differences between the peduncular and terminal sectors. Some peptides may coexist in the same neurons (Swanson, 1987). OXT and GAL, e.g., co-localize in neurons of the adult paraventricular and supraoptic nuclei (Landry et al., 1991; see Rossmanith et al., 1996; Foradori et al., 2006; Furutani et al., 2013, and Bartzen-Sprauer et al., 2014 for other examples). As regards its molecular profile, this domain co-expresses *Otp* and interactive *Sim1*/*Arnt2* genes, within a *Pax6*-positive and *Dlx/Arx/Shh/Nkx2.1*-negative background (Figure 1C; Puelles et al., 2012). Mice lacking *Otp* or *Sim1*/*Arnt2* functions lost the differentiation of OXT, AVP, SST, CRH, and TRH neurons at the paraventricular and supraoptic nuclei, as well as at the RPa (Michaud et al., 1998; Acampora et al., 1999; Wang and Lufkin, 2000; Michaud, 2001; Caqueret et al., 2005). Unfortunately, *Gal, Penk, Cartpt* and *Ghrh* cell types were not studied in these reports. *Ghrh* cells possibly were disregarded because in the adult this phenotype largely seems restricted to the arcuate nucleus. However, *Ghrh* cells separately originate at the basal Arc area, and the alar peduncular Pa, whose derivatives secondarily invade massively the basal plate (Figure 2; Morales-Delgado et al., 2014). In *Otp* null mice, *Ghrh* cells were apparently absent in the basal areas that receive tangentially migrated alar *Ghrh* elements, while the intrinsic *Ghrh* cells at the tuberal Arc were not affected.

The topographic and rostrocaudal distribution of *Trh-, Ghrh-, Cartpt-, Sst-, Avp-, Penk, Crh*-, *Oxt-* and *Gal*-expressing cells within the Pa highlights dorsoventral microzonal differences, which are most evident at the main peduncular Pa (DPa, CPa, VPa; TPaD, TPaC, TPaV; Figure 2). The earliest differentiated paraventricular *Trh, Ghrh, Cartpt, Sst, Avp* and* Penk* cells are circumscribed to the VPa/TPaV subdomains, whereas the earliest *Crh, Oxt* and *Gal* cells appear restricted to the CPa/TPaC subdomains (Figures 3A,B,F,G,I,K,L,P,V; Table 2; see also Morales-Delgado et al., 2011, 2014). Curiously, none of the analyzed peptidergic populations arises primarily in the DPa/TPaD subdomains, though these are secondarily invaded by some of them. A rostrocaudal peculiarity of the distribution of these paraventricular cells is that the peduncular populations (CPa, VPa sources) largely remain close to the ventricle, forming the *Pa* complex (Figures 3I,J,L,N,O,U–W,Y), though there are relatively more superficial elements in the LPa nucleus (radially superficial to VPa) and in the *dorsal entopeduncular nucleus* (EPD; interstitial within the cerebral peduncle) (Figures 3F,G,S). In contrast, the terminal paraventricular derivatives (TPaC, TPaV sources) largely eschew the deep RPa (aPV), excepting the *Sst* cells, which are abundant there, and mostly aggregate instead within the subpial *terminal supraoptic nucleus* (TSO; Figures 3G,H,J’,K,M,X). Others incorporate into a conspicuous cell stream that migrates early on into the tuberal area, forming the subpial *tuberal suboptic nucleus* (TuSbO; this new term corrects the misleading one “tuberal supraoptic nucleus”; Puelles et al., 2012), and the related deeper *tuber cinereum* (TCi) cell population (Figures 3H,J,L,M,X; see TuSbO and TCi in Puelles et al., 2012; their Figures 8.27, 8.32, 8.33).

*Trh* cells first appear in the VPa at E10.5, and later are found also in the TPaV (E12.5), as well as in the CPa and DPa subnuclei. In the adult rat *Trh* cells were recently described likewise in the tuberal lateral hypothalamus (Horjales-Araujo et al., 2014). *Ghrh* cells also have early E10.5 origins in the VPa and appear in the TPaV at E12.5 (Table 2; Morales-Delgado et al., 2014). Some of these *Ghrh* and *Trh* elements then move tangentially ventralwards into basal territories, such as the PBas/ABas, the TuSbO nucleus, and the shells of the VM and DM-P nuclei (Morales-Delgado et al., 2014; their Figure 13). *Cartpt* cells were first identified at VPa at E11.5 (where they persist at P4) and appeared at the TPaV at E13.5 (Figures 3A,B; Table 2). Some peduncular elements disperse into neighboring domains underneath, such as the alar PSPa and the basal PBas (compare Figures 3A,C), while terminal elements seem to migrate into the alar TSPa (Figures 3B, 5D). In newborn mice, *Cartpt*-expressing cells were described as associated to *Lhx6* expression in a population at the ventral part of the PSPa (Shimogori et al., 2010); these might correspond to our migrated *Cartpt* elements.

*Sst* cells emerge within VPa at E12.5 and within TPaV at E13.5, where they largely remain visible, though the deep elements of the terminal RPa possibly disperse partly into TPaC and TPaD (Morales-Delgado et al., 2011; *Allen Developing Mouse Brain Atlas*). Singularly, *Avp* cells were first observed at the superficial LPa/EPD nuclei (peduncular Pa domain) at E13.5; deeper cells appear at the VPa and TPaV at E15.5 (Figures 3F–I; Table 2). Later, *Avp* cells move from VPa into CPa, where they subsequently predominate (Figures 3J, 5C; *Allen Developing Mouse Brain Atlas*), and from the TPaV (E15.5) into the shell of the suprachiasmatic nucleus (SCH) and the TuSbO; in this case, *Avp* cells nearly disappear from their ventral paraventricular sources postnatally (Figures 3H,J,J”, 5C; Table 2). *Penk* cells likewise appear in VPa at E13.5 and TPaV at E18.5 (Figures 3P,R); the VPa ones remain at this location thereafter, whereas the TPaV counterparts seem to invade the SCH shell (Figures 3S,T, 5E).

On the other hand, among the peptidergic populations originated at the central portion of the peduncular Pa,* Crh* cells were first detected there at E13.5 (Morales-Delgado et al., 2014), whereas *Oxt* and *Gal* cells made their earliest appearance at E15.5 (Figures 3L,V; Table 2). At the usually retarded TPaC area, similar *Oxt* cells emerge at E15.5 and *Gal* cells were seen at E18.5. From E18.5 onwards some peduncular *Oxt* cells appear also in the VPa (and the LPa cell group, which appears radially displaced relative to the VPa), and other cells seem to enter the DPa (compare Figures 3L,N,O, 5A); terminal *Oxt* cells become ventrally displaced into the SCH shell and the TuSbO nucleus (Figures 3L,M,O, 5A), jointly with some terminal *Gal* cells (Figures 3X, 5B). *Oxt* cells also appeared at the TSO at E15.5, while a less dense *Gal* cell population was found there from E18.5 onwards (Figures 3K,M,X, 5A,B; Table 2; Jing et al., 1998). The body of terminal paraventricular cells migrating into the TuSbO is observable as a characteristic cellular arch passing dorsoventrally deep to the optic tract, extending from the alar TSO into the basal TuSbO between E11.5 and E13.5. We only illustrate it for *Avp* cells (Figures 3H, 5C). In the adult hypothalamus, *Gal* and *Crh* transcripts in mice (and CRH peptide in rats) are still concentrated mainly at the CPa, with a minority of cells dispersed into VPa and DPa and the cited basal areas; this indicates that migratory dispersion of these elements is limited (*Allen Developing Mouse Brain Atlas*; compare Figure 10 of Swanson, 1987, and Figure 4 of Simmons and Swanson, 2009; note these authors use the old columnar axis ending in the telencephalon; they accordingly describe as “rostral” and “caudal” our prosomeric “dorsal Pa” and “ventral Pa” positions; Puelles et al., 2012).

We mentioned above that the transcriptional functions of* Otp* and *Sim1/Arnt2* are required for the development of at least some of these paraventricular peptidergic neuron types (Michaud et al., 1998; Acampora et al., 1999; Wang and Lufkin, 2000; Michaud, 2001; Goshu et al., 2004). However, the partially overlapping domains of expression of these genes across the peduncular and terminal Pa area do not seem sufficient to explain the above described dorsoventral dissociation of central and ventral paraventricular progenitor subdomains in the paraventricular complex. Some of these differential effects can perhaps be attributed to the *Brain-2* (*Brn2*), and *Sim2* genes, which seem likewise involved in the differential specification of peptidergic cell lineages in this region. See below for data on *Sim2*. The *Brn2* and *Sim1* expression domains selectively overlap within the CPa domain at E12.5 and E13.5, whereas VPa only expresses *Brn2*, and DPa only displays *Sim1* signal (Figure 7 of Michaud et al., 1998; note their columnar rostrocaudal axis again corresponds to our prosomeric dorsoventral axis).

The reported observation that *Cartpt* cells are diminished in number in *Sim1*^−/−^ mice at E12.5 (Caqueret et al., 2006) is difficult to understand, since these cells apparently originate at the VPa, where *Sim1* is not expressed. Moreover, in newborn mice, BRN2 protein co-localizes with *Crh*-, *Avp*- and *Oxt*-expressing cells, which occupy CPa, but not significantly with *Trh* cells, which are produced at the selectively *Brn2*-expressing VPa area (Schonemann et al., 1995; Morales-Delgado et al., 2014). These two apparently inconsistent results may be explained by our earlier (Morales-Delgado et al., 2014) and present data indicating that the *Trh* and *Cartpt* cells born at the VPa normally migrate into the retrotuberal basal plate. The reported absence of *Cartpt* cells in the Pa complex of *Sim1*^−/−^ mice (Caqueret et al., 2006) may be spurious, if these cells were produced normally (no *Sim1* normally at the VPa), and simply migrated away. The same interpretive error perhaps occurred with *Trh* cells with co-localized BRN2 protein, which probably were not searched for where they lie *after migration*. Moreover, in *Brn2-*null mice, no *Crh, Avp* and *Oxt* cells were detected in the Pa and TSO nuclei, while the *Sst* and *Trh* mRNA expression in VPa/TPaV was unaffected (Schonemann et al., 1995).

Conversely, *Trh* and SST cells coincide with the alar expression domain of *Sim2*, which is largely restricted to VPa/TPaV, while there is no topographic correlation between the *Sim2*-expressing domain and *Crh-, Avp-* and *Oxt-*positive cells in neonatal mice (Goshu et al., 2004). As expected, the number of *Trh* and SST cells was reduced in *Sim2* null mice, whereas *Crh*-, *Avp-* and *Oxt*-expressing cells were not affected (Goshu et al., 2004).

In summary, the development of *Crh, Oxt* and *Avp* cells within CPa seems to occur under control of *Otp* and *Sim1/Arnt2*, probably associated to maintained *Brn2* expression. In contrast, *Trh* and *Sst* cells, and probably also *Cartpt* cells, emerge in the VPa/TPaV, controlled by overlapping signals of *Otp, Brn2* and *Sim2* (in absence of *Sim1*). Unfortunately, no experimental data are available with respect to the possible defects in the distribution of alar *Ghrh, Cartpt, Penk* or *Gal* cells in the *Otp, Sim1, Sim2* and *Brn2* mutants.

Additional spatially restricted signals possibly are also implicated in the fate determination of these and other peptidergic cell lineages in the paraventricular area. For instance, CPa strongly expresses *Dickkopf 3* (*Dkk3*) from E11.5 onwards (see *Allen Developing Mouse Brain Atlas*), making this transcription factor another candidate bearing on the differentiation of CRH, galanin and/or OXT cells.

### Alar hypothalamic sources: the subparaventricular area

The subparaventricular domain (TSPa/PSPa; Puelles et al., 2012) is primarily negative for *Otp*/*Sim1*, and positive for *Dlx, Arx, Isl1, Lhx6* and *Vax1* (Figure 1C; Shimogori et al., 2010; Puelles et al., 2012); the early expression of *Arx* is subsequently downregulated, whereas *Dlx* family signals remain expressed into perinatal stages, associated to the differentiation of gabaergic neurons in the entire domain (Hallonet et al., 1998; Shimogori et al., 2010; Puelles et al., 2012; *Allen Developing Mouse Brain Atlas*). This domain produces the classic anterior hypothalamus (massively developed at the TSPa, compared to the thin PSPa); the derivatives include the periventricular subparaventricular nucleus, the anterior hypothalamic nucleus, and the acroterminal suprachiasmatic nucleus (Figure 1B; Puelles et al., 2012). The latter selectively shows *Vip*-, *Avp-* and *Oxt-*expressing cells (Figure 2), detected from E18.5 onwards (SCH; Figures 3D,E,J,J’,O; Table 2; *Allen Developing Mouse Brain Atlas*). Jing et al. (1998) and VanDunk et al. (2011) reported *Avp* mRNA at the mouse SCH already at E16.5 and E17.5, respectively. At P4, *Avp* and *Oxt* cells were more abundant than *Vip* cells, the latter being restricted to a superficial SCH locus (SCHs; Figure 3T). In the adult, VIP cells largely aggregate at the gabaergic SCH core (Moore and Speh, 1993; Castel and Morris, 2000), while the VP/OXT cells lie within the glutamatergic SCH shell (Silverman and Pickard, 1983; Sofroniew, 1985; VanDunk et al., 2011; Puelles et al., 2012; *Allen Developing Mouse Brain Atlas*).

Due to its acroterminal topography, the SCH primordium is distinct from its nearby subparaventricular neighbor, the anterior hypothalamic nucleus, in that it selectively expresses *Six3, Six6, Rorα* and *Lhx1*, a profile that persists in the adult mouse SCH, with *Rorα* in the shell and *Lhx1* in the core; transient selective expression of* Nkx6.2, Fzd5* and *Rx* occurs as well in this area (Conte et al., 2005; Shimogori et al., 2010; VanDunk et al., 2011; Puelles et al., 2012). Differential programming of neurogenesis at this rostral SPa locus is therefore to be expected. *Rorα* signal progressively becomes restricted to the SCH shell postnatally, with a distribution overlapping that of *Avp/Oxt* cells. However, neither *Avp* or *Vip* expression was affected in *Rorα* null mice (VanDunk et al., 2011). With respect to *Six3*, this gene first appears widely expressed in the rostral neural plate, down to the prospective isthmo/mesencephalic border (Oliver et al., 1995; Bovolenta et al., 1998; Kobayashi et al., 2001; Sánchez-Arrones et al., 2009; Dutra de Oliveira Melo, 2011; VanDunk et al., 2011). Afterwards, *Six3* is progressively downregulated at its caudal end; from E11.5 onwards, *Six3* appears restricted to the terminal hypothalamic prosomere (hp2; Puelles et al., 2012) (*Allen Developing Mouse Brain Atlas*; Dutra de Oliveira Melo, 2011; VanDunk et al., 2011). Complete *Six3* inactivation stunts the whole rostral prosencephalon (telencephalon and hypothalamus; Carl et al., 2002; Lagutin et al., 2003; Lavado et al., 2008). A recent study using a *Nestin*-Cre transgenic line to limit floxed *Six3* loss to neural progenitors produced unexplained variable results at E15.5–E19.5. Some specimens showed absence of *Rorα* and *Avp* expression specifically at the SCH, while *Avp* expression continued to be present in the neighboring RPa and the supraoptic nucleus (TPa derivatives) (VanDunk et al., 2011).

The recent developmental study of VanDunk et al. (2011) clearly represented a significant advance in our understanding of this specialized area. We argue nevertheless that their columnar descriptions would benefit from a translation into prosomeric terms, in so far as prosomeric theory allows fine dorsoventral and anteroposterior regionalization and description of the hypothalamus, whereas columnar theory does not; that is, there is no precise columnar answer to the question ¿which part of the hypothalamic primordium is occupied by the suprachiasmatic nucleus? In contrast, its position within the prosomeric genoarchitectonic map and the framework of possible signaling mechanisms is clearcut in Figure 1B. In spite of the VanDunk et al. (2011) analysis, the cascade of regulators involved in the local differentiation of *Avp* and *Oxt* cell lineages remains unclear (in comparison with the origin of such cells in an *Otp*-expressing molecular background), and so does the mechanism that segregates *Avp/Oxt* cells into the SCH shell (whose glutamatergic profile surprises within the SPa); *Vip* cells unproblematically settle within the SCH gabaergic core. Though VanDunk et al. (2011) concluded there is a single neuroepithelial source for all SCH neurons, experience accrued so far elsewhere in the brain suggests that the same progenitors normally do not produce both glutamatergic and gabaergic cells, leaving apart exceptional observations of neurons displaying both neurotransmitters, whose progenitor mechanisms remain unknown (e.g., Jarvie and Hentges, 2012). Parallel sources for these two components, at the TPa or ABasM, and the TSPa, respectively, should perhaps be considered.

Puelles et al. (2012) conjectured that the vasopressinergic and oxytocinergic cell types (and other glutamatergic cells) of the SCH shell might originate in the suprajacent acroterminal TPa progenitor area, which also expresses *Six3*, and is characterized by *Otp*/*Sim1/Brn2* expression (plausibly a necessary genetic background for these peptidergic phenotypes; note the underlying ABasM area also expresses *Otp* in conjunction with basal markers). According to this hypothesis, prospective* Rorα, Avp* and *Oxt* shell components might migrate tangentially at early stages from the acroterminal TPa into the subjacent SCH primordium within TSPa (Figures 5A,C). *Rorα* expression is most dense next to the TPa/TSPa boundary in sagittal sections, and the early *Rorα* cells form a marginal stratum covering early *Lhx1* cells, supporting such a nearby source and migration mechanism (Figures 2B, 4 of VanDunk et al., 2011). The variant hypothesis may be contemplated that prospective SCH *Avp/Oxt* cells are produced within the TPa, and a combination of *Six3* with specific SPa markers (*Dlx genes, Arx, Isl1, Vax1*) and/or selective SCH regional markers (*Six6, Rorα, Lhx1, Nkx6.2, Fzd5, Rx)* defines a SCH domain that selectively *attracts* these *Avp/Oxt* cells from the TPa into the incipient SCH shell subregion (question marks in Figures 5A,C).

### Basal hypothalamic peptidergic cell sources

As mentioned above, the basal hypothalamus is primarily divided dorsoventrally into the tuberal/retrotuberal (Tu/RTu) and the primary mamillary/retromamillary (M/RM) regions (Figure 1). The Tu/RTu territory is subdivided into dorsal, intermediate and ventral subdomains (TuD/RTuD; TuI/RTuI; TuV/RTuV; Puelles et al., 2012). The TuD/RTuD produces glutamatergic cells for the periventricular anterobasal and posterobasal nuclei (ABas, PBas), plus some intermediate populations such as the nucleus of the TCi, the magnocellular lateral hypothalamic nucleus (MCLH), and several ventralwards migrating populations, including the massive ventromedial nucleus (VM; Puelles et al., 2012); ABas can be further subdivided into acroterminal/median—AbasM- and wing—AbasW- portions. Leaving apart the migrated ventromedial nucleus, the TuI/RTuI contains intrinsic gabaergic populations belonging to the terminal and peduncular parts of the dorsomedial nucleus* shell domains* (DMsT, DMsP), as well as to the acroterminal arcuate nucleus *shell* (Arcs); these formations all have glutamatergic *core* cell aggregates whose presumptive migratory origins are under study (Puelles et al., 2012). In addition, there exists the glutamatergic ventral premamillary nucleus, which migrates from the retromamillary area into DMsT (VPM; Figure 1B; Puelles et al., 2012). The remaining thin TuV/RTuV domain is related to production of the hypothalamic histaminergic neurons (Puelles et al., 2012). On the other hand, the primary M/RM area subdivides into the *Otp/Sim1*-positive perimamillary/periretromamillary band, and the secondary M/RM complex proper, both of which produce exclusively glutamatergic neurons.

### Basal hypothalamic sources: the dorsal tuberal and retrotuberal areas

The acroterminal part of the anterobasal area (ABasM; TuD), where *Six3* is selectively expressed, develops an early *Otp*-positive cell population, some of whose elements differentiate into diverse peptidergic cell types (e.g., Morales-Delgado et al., 2011). Jointly with the peduncular RTuD, the TuD subdomain is characterized molecularly by the expression of general basal markers such as *Shh* and *Nkx2.1*, and more restricted expression of other markers such as *Vax1, Lmo3, Enc1, Vat1l*, and *Cnr1* (Figure 1C; *Allen Developing Mouse Brain Atlas*). A subliminal dorsal section of this domain also expresses *Nkx2.2* (Puelles et al., 2012; their Figure 8.14B).

The first peptidergic cell types detected at the ABasM are *Sst* and *Pomc* cells, first detected at E10.5 and E11.5, respectively (ABasM; Figure 4A; Table 2; present data; McNay et al., 2006; Morales-Delgado et al., 2011). *Cartpt-, Penk*- and* Npy-*expressing cells next appear throughout ABas at E13.5 (ABasM and ABasW; TuD; Figures 3B,P, 4D; Table 2), whereas *Pmch* cells first appear at E11.5 at the PBas (RTuD; not shown; see *Allen Developing Mouse Brain Atlas*; Croizier et al., 2011), and *Sst, Penk, Npy* and* Hcrt* cells next emerge there between E13.5 and E15.5 (Figures 4D,H; Table 2). The peptidergic cell types mentioned at the ABas differentiate as subtypes within the preexistent abundant population of *Otp/Nkx2.1*-positive postmitotic neurons, whereas, in contrast, the peduncular PBas area contains few *Otp/Nkx2.1* cells (Figures 1C, 2; Morales-Delgado et al., 2014). At subsequent stages, peptidergic cells like those of the ABas appear to invade the subjacent tuberal (acroterminal) arcuate nucleus, as well as the shell of the migrated VM nucleus, which also originates from the TuD area (Figures 5D–G; Morales-Delgado et al., 2011; Puelles et al., 2012). As is further discussed below, we conclude that ABas is the source of these topographically tuberal *Sst, Cartpt, Penk, Pomc*, and *Npy* cells (which also are accompanied by migrated *Otp*-positive cells). In *Otp*-null mice, cells expressing *Sst* mRNA or producing SST peptide were absent in the Arc and in an “adjacent area”, probably the VM shell (Acampora et al., 1999; Wang and Lufkin, 2000). It would be interesting to analyze the other peptidergic cell populations originated at ABas/PBas in these mice, in order to check their dependency on *Otp* expression and function.

The ABas domain expresses additionally *Nr5a1* (*SF1*) at E9.5–E11.5 (Figure 7 of Ikeda et al., 2001), coinciding with the appearance of the earliest *Sst* and *Pomc* cells (Table 2; Shimogori et al., 2010; Morales-Delgado et al., 2011). SF1- and POMC-immunoreactive neurons overlap at the acroterminal ABasM at E10.5 (Figure 1C of McNay et al., 2006; these authors identified it as “retrochiasmatic nucleus”, which is a classic synonym for ABas). Subsequently, at 13.5, *Nr5a1* (*SF1*) labels also the AbasW, as well as a VM subpopulation that moves massively ventralwards into TuI (see *Allen Developing Mouse Brain Atlas*; Puelles et al., 2012). Separately, *Pomc* labels since E12.5 a parallel dorsoventral migration from the acroterminal ABasM into the acroterminal Arc area (Figures 4B,C, 5G; Shimogori et al., 2010; Puelles et al., 2012;* Allen Developing Mouse Brain Atlas*). These migrations thus start selectively at the dorsal tuberal ABasW and ABasM subareas, respectively. A number of *Cartpt, Sst*/*Otp* and *Npy* cells accompany both migrations, and thus reach as a common terminus the VM and Arc shell regions (TuI; Figures 3C, 4D’,E–G, 5D,F; Morales-Delgado et al., 2011). In murine *Nr5a1* knockout mutants, the distribution of cells expressing NPY, GAL and estrogen receptor α was altered, but the differentiation of these phenotypes was unaffected (Dellovade et al., 2000). The VM nucleus itself appeared subtly altered in this mutant, with partial fading of its normally sharp boundary, allowing some intermixing of peripheral GAD67 cells, as well as disgregated GFP-labeled SF1−/− neurons and redistribution of other components (e.g., ISL1, BDNF, NKX2.1, NPY immunoreactive cells; Dellovade et al., 2000; Tran et al., 2003; Davis et al., 2004). On the whole, these studies suggest that the final topography of the diverse peptidergic or non-peptidergic neuronal types within and surrounding the VM is altered as a consequence of the dismorphogenesis caused by *Nr5a1* mutation. It may be speculated that other peptidergic cell types originated at the ABas may be also partially relocated in the *Nr5a1*-null phenotype.

In the adult rat, NPY cells are generally ascribed to the Arc, plus transient *Npy* expression at other sites. Our data in the mouse indicate there is in addition early appearance of *Npy* cells within the ABas and PBas areas at E13.5 (Figures 4D,D’). Subsequently some of these *Npy* cells migrate into the intermediate tuberal and retrotuberal areas, targeting the Arc and DM nuclei, and the shell of VM (Figures 4E–G, 5F). These migrated elements may correspond with the reported rat dorsomedial, perifornical and lateral hypothalamic *Npy* cells (Singer et al., 2000; Grove et al., 2001).

*Cartpt* cells first appeared at ABas at E13.5, with an apparent later expansion of the source into the peduncular PBas, and subsequent dispersion into terminal and peduncular Tu/RTu regions such as the VM shell and the DM shell (Figures 3B,C, 5D), consistently with Koylu et al. (1997), Broberger (1999), Vrang et al. (1999), and Elias et al. (2001). *Cartpt* cells were first observed at the Arc at P14. We also observed *Cartpt*-positive cells in the mammillary area from P4 to P56, a localization which was not previously reported (M; Figures 3C, 5D; *Allen Developing Mouse Brain Atlas*). This late *Cartpt* cell population may be interpreted either as due to cell dispersion from TuI, with a more dorsal origin (TuD), or as late timing of the expression of the marker.

*Mash1* (*Ascl1*) codes a transcription factor, whose expression generally overlaps and parallels in regional intensity that of *Dlx* genes (SPa ventricular and mantle zones in the alar hypothalamus, and the ventricular zone of the whole basal Tu/RTu region). In the absence of *Mash1* function, a general reduction of cell numbers was observed at the VM and Arc nuclei, affecting the POMC, NPY, GHRH and dopaminergic lineages (McNay et al., 2006). At E10.5 there is already a dramatic reduction in the number of SF1/MASH1 and POMC cells at the ABas, whereas at later stages migrated SF1 (*Nr5a1*) cells in the VM, as well as migrated NPY cells and intrinsic dopaminergic neurons in the Arc are significantly reduced (McNay et al., 2006). As regards the differentiation of GHRH cells at the Arc, see comments below on requirement of *Msh1* and* Gsh1* functions.

The early restricted expression of *Pomc* at the ABasM (acroterminal TuD) without any additional expression at the ABasW and PBas may be related to the local restricted signal of *Six3* (or other genes mentioned above) within the acroterminal area. *Six3* is reportedly necessary for *Shh* activation in the terminal hypothalamus (Geng et al., 2008). General basal *Shh* and *Nkx2.1* expression is necessary for the development of the tuberal hypothalamic regions, though, curiously, the ABas itself is not affected in mice lacking *Nkx2.1* function, whereas the Arc and VM nuclei are absent or abnormal (Kimura et al., 1996). Moreover, the *Neurog3* (*Ngn3*) gene is expressed selectively at ABas as early as E9.5 (described as “Arc/VM region of the hypothalamus” by Pelling et al., 2011). *Mash1* apparently acts upstream of *Ngn3* to regulate neurogenesis in the basal hypothalamus (McNay et al., 2006). Pelling et al. (2011) showed in *Ngn3*-Cre mice that a set of POMC, NPY, TH and SF1 cells originate from *Ngn3* progenitors, presumably at the ABas area. This result suggests that the arcuate TH-positive population of dopaminergic cells may also migrate tangentially down from the ABas domain (an idea never proposed before, but presently corroborated by our finding of ABas *Th*-expressing cells at E11.5 (*Allen Developing Mouse Brain Atlas*). Moreover, loss of *Ngn3* function leads to a significant reduction of POMC cells, combined with an increase of TH and NPY cells (Pelling et al., 2011), implying that this factor promotes the POMC phenotype but represses the NPY and TH fates.

Basal *Pmch* cells were already present superficially at E11.5, restricted to the subliminal dorsal retrotuberal PBas area (not shown; *Allen Developing Mouse Brain Atlas*); we identified this population according to Puelles et al. (2012) as the conventional *magnocellular lateral hypothalamic nucleus* (MCLH; Figure 4L); similar cells were included in the lateral hypothalamus by Croizier et al. (2011). Additional PBas *Pmch* cells later disperse between E15.5 and P4 within the *dorsobasal and ventrobasal sectors of the lateral hypothalamus*, deep to the lateral forebrain bundle (see these sectors in Puelles et al., 2012; their Figure 8.32), deep to the migrated subthalamic nucleus. Some of these elements also invade the DM-P shell and the dorsobasal/ventrobasal perifornical nuclei (MCLH, LH, DBLH, VBLH; Figures 4N–P, 5H; see P56: Figure 4Q).

The *Pmch* cells found in the adult rat lateral hypothalamus, including the MCLH, were labelled by acute BrdU at E10-E11 (Brischoux et al., 2001; Croizier et al. (2011). The latter authors mapped the earliest MCH neurons in rat embryos, compared their positions with relevant regional molecular markers (emphasizing their origin within a *Nkx2.2/Nkx2.1/Shh*-positive band), and studied the course of their efferent axons. We are essentially in agreement with their primary data, but qualify the interpretation given by these authors about the molecular environment of the early *Pmch* cells. This is partly because we use the more elaborated model of molecularly distinct progenitor domains reported by Puelles et al. (2012), which was not available to these authors. Croizier et al. (2011) convincingly showed in E14 rat embryos that the early *Pmch* cells occupy a restricted posterior sector of the classical longitudinal hypothalamic cell cord, which is formed by precociously differentiating basal neurons (Gilbert, 1935; Keyser, 1972; His, 1892; see also Puelles et al., 1987, 2014). Consistently with this interpretation, this band expresses *Shh* and *Nkx2.1* (typical overall basal plate markers), as well as *Nkx2.2* (an early longitudinal marker expressed *across* the alar-basal boundary, thus labeling a subliminal part of the hypothalamic cell cord, as well as a liminal part of the overlying subparaventricular area in the alar plate; Figure 1A; Puelles and Rubenstein, 2003; Puelles et al., 2004, 2012; Figure 8.14B of the 2012 reference). Within our model, the basal subliminal band falls specifically within the TuD/RTuD progenitor area. The restricted *peduncular* position of the early *Pmch* cells, corroborated by our present mouse results, jointly with the molecular environment provided by Croizier et al. (2011), is consistent with a subliminal PBas (RTuD) origin, a site with selective expression of *Lhx9* (Figures 1, 5H; Shimogori et al., 2010).

However, Croizier et al. (2011) assumed that the early basal cell cord is identical with the “intrahypothalamic diagonal” of Shimogori et al. (2010), thinking it correlates with the longitudinal band that expresses *Nkx2.2*. This interpretation seems wrong in two ways. First, the *Nkx2.2* band has alar and basal parts (as contemplated in Puelles et al., 2004, 2012), and the differentiating MCH cells are restricted to the basal component, as was clearly indicated by the overlapping expression of basal markers such as *Shh* and *Nkx2.1* (Croizier et al., 2011; their Figures 3K–P). Second, these authors apparently misinterpreted the description of the molecular profile of the intrahypothalamic diagonal given by Shimogori et al. (2010), insofar as these authors did not identify their diagonal area as expressing *Shh* and* Nkx2.1*. The intrahypothalamic diagonal is the distinct *alar* longitudinal SPa territory that overlies the basal hypothalamic cell cord (TuD/RTuD); it expresses *Dlx*/*Arx*/*Vax1/Isl1* transcripts and some *Lhx* genes, but lacks significant *Shh* or* Nkx2.1* signal (Shimogori et al., 2010; Puelles et al., 2012; Figure 1C). A ventral part of this area—the *liminal* SPa subarea- shows *Nkx2.2* signal on top of the cited molecular profile (Puelles et al., 2012; see their Figure 8.14B). The *Pmch* cells clearly lie under it (Croizier et al., 2011; their Figures 3K–P), within the underlying *Nkx2.1/Nkx2.2*-positive subliminal TuD/RTuD basal domain, which corresponds to the true hypothalamic cell cord.

Hypothalamic *Hcrt/orexin* cells also differentiate selectively within the peduncular dorsal retrotuberal region, though later, at E15.5 (Figures 2, 4H). We think that this occurs specifically within the ventral PBas subarea that does not express *Nkx2.2* (present data). Progressively, most *Hcrt/orexin* neurons disperse radially and tangentially into the (retrotuberal) *ventrobasal sector of the lateral hypothalamus*, where *Nkx2.2* is not expressed (VBLH; Figures 4H,I, 5I; compare Figure 8.29 of Puelles et al., 2012), though some remain more dorsally, within their source area PBas. In the adult rat brain, *Hcrt* cells were found just rostral to the zona incerta, within the “lateral and dorsal hypothalamus” and the “perifornical nucleus” (Peyron et al., 1998; their Figures 3, 8 and 14; compare Figure 1). This location lies just dorsally to our PRM area, pinpointing the RTuI, or ventrobasal, part of the LH. The singularity of PBas as a cell source, as opposed to the terminal ABas domain, might be due to its distinctive genoarchitectonic properties, such as the lack of *Nr5a1* expression (Puelles et al., 2012; present data) and the coincidence with selective *Lhx9* expression (Shimogori et al., 2010). The latter authors reported that *Lhx9* is co-expressed with *Hcrt* and partially also with *Gal* cells in newborn mice. In conditional *Shh* mutant mice in which *Shh* was selectively abolished in the basal hypothalamus, the entire Tu/RTu region was very reduced, and* Hcrt* cells were not detected (Szabó et al., 2009); on the whole, this supports our conclusion of a restricted *basal Hcrt* source at the ventral part of PBas. In contrast, Zhao et al. (2008) suggested an alar prethalamic origin of at least some *Hcrt* cells, based on the presence of a few LH hypocretin /orexin-expressing cells that coincide with *Foxb1-*derived progeny in newborn mice. This possibility needs additional analysis, since it postulates both a caudorostral (prethalamo-hypothalamic) and dorsoventral (alar-basal) translocation of such cells, which would need to be distinguished from alternative *Foxb1-*derived progeny potentially produced at the basal mamillary domain, some of which move into periretromamillary positions, just under the VBLH (Zhao et al., 2008).

### Basal hypothalamic sources: the intermediate tuberal and retrotuberal areas

This section centers on the arcuate and dorsomedial domains. The tuberal arcuate nucleus, an acroterminal basal locus characterized by overlapping *Nkx2.1*/*Dlx/Six3* markers, combined with early downregulation of its initial basal *Shh* expression (Manning et al., 2006), is an important source of some peptidergic neurons, such as *Ghrh* (Morales-Delgado et al., 2014) and *Agrp* cells (present data). We postulated above that the adult local dopaminergic (*Th*), and *Pomc* cell types originate more dorsally at the *Ngn3-*expressing ABas domain (ArcM, ArcW; Figure 5G; Pelling et al., 2011). It is less clear whether *Npy* cells are included in the same migratory pathway. Such cells do appear first at the ABas area, and later at the Arc. It was established that most adult POMC cells are not gabaergic cells (consistently with the postulated ABas origin), whereas a majority of NPY neurons are gabaergic, which suggests instead a local Arc origin, though they may be complemented with some migrated cells (Horvath et al., 1997; Ovesjö et al., 2001; Hentges et al., 2004; Puelles et al., 2012). Significantly, about one quarter of the mature NPY neurons in the Arc share a common progenitor with POMC cells (Padilla et al., 2010). Accordingly, two subgroups of Arc NPY cells probably exist: 25% is glutamatergic, originated at the *Ngn3*/*Nr5a1/Six3*-positive ABas locus, and a majority (75%) gabaergic population is generated within the Arc, in clear correspondence with local *Dlx* and *Gad67* expression (Yee et al., 2009; Puelles et al., 2012; Figure 5F).

Dispersed *Ghrh* cells first appear at E13.5 at the wing (non-acroterminal) part of the arcuate nucleus, extending later into the median acroterminal Arc (ArcM) where they form an important *Ghrh* cell population (ArcW, ArcM; Table 2; Morales-Delgado et al., 2014; *Allen Developing Mouse Brain Atlas*). On the other hand, *Agrp* cells first develop at the median part of the arcuate nucleus (ArcM) somewhat later, from E15.5 onwards. *Agrp* cells thereafter remain fully restricted to the Arc during postnatal development (Figures 4J,K; Nilsson et al., 2005). Some of the genes expressed at the Arc locus, such as *Six3, Nkx2.1, Mash1, Hmx2, Hmx3, Gsh1, Aldh1a2*, and *Isl1* were implicated in the differentiation of GHRH cells. *Nkx2.1* is expressed throughout the mantle of the hypothalamic basal plate at prenatal stages, and conditional *Nkx2.1* mutants are defective in the Arc derivatives (Mastronardi et al., 2006). The NKX2.1 transcription factor acts upstream of *Mash1, Hmx2*/*Hmx3* and *Gsh1* (Caqueret et al., 2005). *Mash1* and *Hmx2*/*Hmx3* control of *Gsh1* expression, which is required for the specification of GHRH neurons (Wang et al., 2004; McNay et al., 2006). Unfortunately, these studies did not explore the role of those genes in the differentiation of *Agrp* cells. Interestingly, *Agrp* cells were unaffected in null mutants of *Ngn3* (Arai et al., 2010), a transcript restricted to ABas, consistently with their apparent Arc origin.

Caudal to the Arc, the intermediate Tu/RTu subregion shows the mutually similar DM-T and DM-P areas, which share *Shh, Dlx, Nkx2.1, Isl1, Cnr1, Peg10* and *Lef1* expression, in absence of *Lhx9/Lhx6* signals (DM-T, DM-P; Figures 1B,C). Both DM portions (as well as the Arc) are subdivided into core (glutamatergic) and shell (gabaergic) portions; the core cells were suspected of migrating into these areas from a neighboring origin, probably the TuD/RTuD, whereas the gabaergic shell elements are intrinsic, consistently with local *Dlx* family genes and *Gad67* (Puelles et al., 2012).

Interestingly, the DM-P domain is a major source of *Gal* cells; the latter were first identified there at E15.5 (Figure 3V; *Allen Atlas* data). Similar early *Gal* cells were reported to appear in a vaguely defined “precursor hypothalamic region” at E16 in the rat, which we estimate to be peduncular and retrotuberal (RTuI) in topography (Figure 1A of Gundlach et al., 2001). Starting at E18.5, a less important *Gal* population appears likewise at the DM-T area, jointly with a possibly migrated perimamillary subpopulation (not shown; see *Allen Atlas* data). Postnatally these cells concentrate at the DM *shell* domains (Puelles et al., 2012), a pattern that suggests a local origin within TuI/RTuI and a gabaergic nature (DMsP, DMsT, Figures 3V,W,Y,Z, 5B; Table 2; Puelles et al., 2012). Additional *Gal* cells appear at the Arc and the ventrobasal lateral hypothalamus postnatally (VBLH; Figures 3Z, 5B; compare Melander et al., 1986). This suggests partial migratory dispersion of DM-P/DM-T *Gal* cells into the PM, Arc and VBLH areas (Figure 5B).

### Basal hypothalamic sources: the perimamillary and periretromamillary areas

Another basal progenitor domain producing peptidergic cell types is the arc-shaped perimamillary/periretromamillary area (PM/PRM), which lies ventral to the linear *Nkx2.1*/*Dlx*/*Arx*/*Lhx6/Wnt8b*-expressing TuV/RTuV domain (tuberomamillary terminal of Shimogori et al., 2010), where histaminergic neurons selectively originate (Puelles et al., 2012; Figure 1C). The PRM/PM area represents a domain with precocious neurogenesis, comparable in this regard to the hypothalamic cell cord (TuD/RTuD; see Puelles et al., 2014). It is molecularly distinct from the retarded retromamillary and mamillary areas by its selective expression of *Otp* and* Sim1*, in curious parallelism with the alar paraventricular area (Figure 1C; Puelles et al., 2012). *Sst, Penk, Npy* and *Pmch* phenotypes seem to originate from both the peduncular (PRM) and terminal (PM) subdivisions of this domain, in each case with a particular temporal sequence (Figure 2; Table 2; Morales-Delgado et al., 2011). It is unclear whether the PM/PRM *Gal* cells interpreted above as migrated from the DM-P area might originate instead locally (this point can be decided by examining their gabaergic vs. glutamatergic nature, since PM/PRM produces only the latter type; Puelles et al., 2012). *Sst* and *Npy* cells appeared selectively at the PRM at E13.5 and were detected only at E18.5 and P4, respectively, within the PM; this delay raises the possibility of their tangential translocation from PRM into PM (Figures 4D–G, 5F; Table 2; Morales-Delgado et al., 2011; Morales-Delgado, 2012). *Penk* cells were not found at E15.5, but were already well represented at both PRM and PM at E18.5 (Figure 3R), which suggests an earlier appearance. At the later stage, as well as postnatally, the *medial PRM nucleus* (PRMM; Figure 3R) and the *dorsal premamillary nucleus* (DPM; Figure 3R; this is the main PM derivative) show massive *Penk* cell populations, and additional *Penk* cells were found at a previously undescribed rounded perimamillary cell aggregate superficial to the DPM, named here *superficial perimamillary nucleus* (PMS; Figure 3U; see also the Allen Developing Mouse Brain Atlas, transversal P56 series [64881286], section 61, for DPM and PMS). The neighboring *ventral premamillary nucleus* (VPM) also contains *Penk-*positive cells; however, it lies in the TuI area, intercalated between the ventromedial nucleus and the PM band (not shown; see the P56 Allen image cited above). Though these tuberal cells might have a different, more dorsal basal origin (see above results on ABas/PBas *Penk* cells), we mention them here because of the possibility that these VPM cells may have migrated either from the underlying PM area or from the more distant PRM area, accompanying the migration of RM cells into VPM (Figure 5E; Puelles et al., 2012).

Medial hypothalamic *Pmch* cells were also clearly identified along the characteristic PRM/PM band from E13.5 onwards (Figures 1, 2, 4M–P; Table 2). A neighboring *Pmch* cell group forms a shell around the migrated ventral premamillary nucleus (TuI area) from E15.5 onwards (VPM; Figures 4N–P). Remarkably, at postnatal stages few *Pmch* cells remain visible at the PRM/PM locus, suggesting an earlier translocation into the suprajacent TuI/RTuI areas, mainly the DM-P and DM-T nuclei, as well as the mentioned VPM shell neighborhood, where they increasingly become visible (Figures 4N–P, 5H). The PRM/PM *Pmch* cells seem unrelated to the more dorsal and precocious ones described above, which contribute to the MCLH and LH populations (see Figures 4Q, 5H). While Croizier et al. (2011) did not identify this separate set of PM/PRM MCH cells, (Brischoux et al. (2001); their Figure 5E; compare with our Figure 1) showed a drawing of a sagittal section, in which rat *Pmch* cells at E18.5 clearly occupy a band that does not agree in shape and position with any part of the basal hypothalamic cell cord (TuD/RTuD), but agrees with our PRM/PM area.

*Otp* and *Sim1*/*2* are potential key genes in the differentiation of the mentioned PRM/PM peptidergic lineages, since they are selectively co-expressed very early at this locus (supplementary figures of Shimogori et al., 2010; Morales-Delgado et al., 2011, 2014; Puelles et al., 2012; *Allen Developing Mouse Brain Atlas*); however, this hypothesis was not tested so far. Other genes expressed at the PRM/PM domain, such as *Shh, Nkx2.1, Pou3f3 (Brn1), Ebf3 and Peg10* (Figure 1C), might be also involved in the neurogenesis and/or differentiation of the peptidergic cell types originated in this territory.

### Prodynorphin progenitor areas

We also studied *Pdyn* cells, which may be a singular case of delayed appearance of the peptidic marker expression in neurons that previously migrated tangentially. *Pdyn* cells first appear at E13.5 within the alar VPa area (peduncular hypothalamus); later they invade the overlying CPa and DPa areas at E15.5 and E18.5, respectively (Figures 4R–U, 5J; Table 2). Basal *Pdyn*-expressing cells were first observed at the DM-P and migrated (terminal) VM from E13.5 onwards, and at the DM-T at P4 (Figures 4R’,S–U, 5J). At perinatal stages, the basal elements became progressively concentrated within the core domains of these nuclei, known to be glutamatergic (e.g., VMc; DMcT in Figures 4T,U). Puelles et al., 2012 argued that there might be a relationship of *Pdyn* cells with *Nkx2.2*-expressing immature migrating elements, which all arise from the longitudinal band mentioned above, which overlaps the alar-basal boundary; this band divides into a *liminal* alar sub-band and a *subliminal* basal sub-band, whose derivatives co-express differential alar vs. basal gene markers (Figures 1A,C, 2). Puelles et al. (2012) showed that, from E10.5 onwards, peduncular liminal *Nkx2.2*-positive neurons selectively migrate dorsalwards into the VPa nucleus, from where some of them later move into the LPa, CPa and DPa nuclei. This sequential pattern recalls our present data on early peduncular *Pdyn* cells, though these are first detected at the VPa at E13.5, and not at the *Nkx2.2* band (Table 2). If they are identical with the earlier migrating *Nkx2.2*-positive cells, then they apparently start to express *Pdyn* in a delayed manner, after their initial migration into VPa ends. In that case, their proper origin would not be the VPa area, but the underlying liminal PSPa subarea (Figure 5J). Co-expression and lineage studies are needed to resolve this issue; *Nkx2.2* continues being expressed in the paraventricular complex even in the adult mouse (Puelles, unpublished observations). It is also relevant to check the potential gabaergic vs. glutamatergic phenotype of these *Pdyn* cells, since local VPa cells are expected to be glutamatergic, whereas PSPa cells are held to be gabaergic (Puelles et al., 2012, note their Figure 8.18 shows gabaergic cells at the VPa and DPa nuclei).

On the other hand, terminal subliminal (basal) *Nkx2.2*-positive cells (from the upper TuD area) migrate between E13.5 and E15.5 ventralwards into the VM nucleus, constituting one of its diverse migrated subpopulations (Puelles et al., 2012; their Figure 8.26 and text; Figure 5J). In this case, we see *Pdyn* cells emerging at the VM locus at E13.5 (Figure 4R’), possibly representing migrated subliminal *Nkx2.2* cells that again express the peptidic marker in a delayed manner, after migration. This interpretation is supported by the fact that all other known populations of the VM migrate from the overlying TuD area. Accordingly, the latter may be suspected to be the true origin of these VM *Pdyn* cells.

The *Pdyn* cells emerging at the DM-P may correlate likewise with peduncular subliminal *Nkx2.2*-positive cells (from the upper RTuD area), since some of them also migrate ventralwards a short distance into the DM-P area at E14.5 (Puelles et al., 2012; their Figure 8.26F). This is where we saw *Pdyn* cells at E15.5, possibly again due to delayed differentiation (Figure 4S; Table 2). Unfortunately, the Allen Atlas lacks clearcut data on *Nkx2.2* expression at this locus at later embryonic stages (few labelled cells appeared at the DM-P locus at E18.5), or postnatally (no significant signal). Contrarily, *Pdyn* cells become increasingly visible postnatally at the TuI/RTuI area, largely aggregated within the developing core portions of the DM-P and DM-T nuclei, as shown here at E18.5 and P4 (Figures 4T,U, 5J; note that due to its obliquity at 45 degrees relative to the ventricle, the DMcP appears in more lateral sections than the DMcT, which is parallel to the ventricle**)**. We likewise lack evidence that any *Nkx2.2-*expressing cells invade the TuI area where the DMcT *Pdyn* cells emerge postnatally. In defense of the migratory hypothesis, it may be conjectured that migration of such cells into the DM areas might imply downregulation of *Nkx2.2* before the *Pdyn* signal appears. Lineage studies are thus needed to test our conjecture that *Pdyn* cells derive systematically from either alar or basal *Nkx2.2*-espressing progenitors. Note the potential origin of the DM *Pdyn* cells at the overlying TuD/RTuD area would explain their glutamatergic phenotype within the gabaergic DM shell environment (Puelles et al., 2012).

### Conclusive comments

Table 2 summarizes our findings about the earliest topography of the different hypothalamic peptides, separated into terminal and peduncular progenitor areas. We subsumed under the terminal progenitor domains the rostral specialized acroterminal areas (Figure 1B; Puelles et al., 2012), which nevertheless show some independent behavior (e.g., SCH, Arc, as indicated in parentheses in Table 2).

1. *Peptidergic neurons (PN) originate independently in many areas*: It is readily apparent that peptidergic neurons originate independently in many areas of the terminal and peduncular parts of hypothalamus, across both alar and basal territories.
2. *The same PN type can originate in two or more separate areas*: Importantly, the same peptidergic type (with the caveat that cells expressing a given peptide may represent different cell types if they co-express differentially other markers) can be produced separately in two or more alar and/or basal areas (another caveat is that whenever adjacent areas show the same cell type in a close temporal sequence the issue is raised whether the retarded area was invaded by cells from the other).
3. *Not all progenitor areas generate PN*: In any case, peptidergic cell production doesn’t seem to be a general feature of the whole hypothalamus, since some alar and basal progenitor areas did not produce any of the studied cell types (areas marked with a light gray background; Table 2).
4. *Most PN-producing areas generate several types sequentially*: An interesting feature is that most progenitor areas that do produce peptidergic neurons generate several types over time (left to right in Table 2); however, in some cases this pattern is an artefact of our simplified table, since the varied cell production occurred in distinct subareas; for instance, at the TSPa area, the early *Cartpt* cells appear aligned with the prospective anterior hypothalamic nucleus, whereas the later *Vip, Avp* and* Oxt* cells emerge at the neighboring acroterminal suprachiasmatic nucleus; moreover, the *Avp/Oxt* types have a question mark in Table 2, because we suspect these elements may come from the overlying TPa area (see text above). A sequential pattern nevertheless is truly present in other areas; this implies a general fate regulatory mechanism that is heterochronic and position-sensitive (i.e., at a given locus, different cell types are generated over time); moreover, the same cell type may differentiate sooner or later in different areas.
5. *One single progenitor area can generate simultaneously 2 or 3 types of PN*: A repeated special case is represented by the results indicating *simultaneous* production of two or more peptidergic cell types at given areas, mostly from E13.5 onwards (items marked with a darker gray background; Table 2). We conjecture this implies the existence of an additional salt-and-pepper regulatory mechanism, probably residing in the postmitotic neurons (via mutual lateral inhibition).
6. *No PN migrate from basal to alar*: In general, the basal plate seems to attract several alar populations, whereas there are no cells moving from basal into alar domains. The latter conclusion is consistent with data on *Shh* lineage reported by Alvarez-Bolado et al. (2012), who noted restriction of migrated basal derivatives to the basal plate territory.
7. *The sequential topography of most PN can be parsimoniously explained by the hypothesis of tangential migration*: We grouped in Table 3 the cell types according to their single or mixed alar *vs*. basal origins (this does not exclude common aspects in the respective molecular backgrounds). This table also shows the respective topographies of these cell populations mapped at perinatal stages, highlighting in bold the cell types that apparently moved from alar origins into basal positions, and marking with an asterisk the populations that apparently moved tangentially within their original alar or basal territories. We conclude that most hypothalamic peptidergic populations can be postulated to migrate tangentially, with few exceptions (e.g., *Vip, Agrp* and some *Npy* cells). For clarity, the deduced tangential migrations are each illustrated graphically in Figure 5, indicating the main tangential targets and the respective timings. These migration hypotheses will no doubt be subjected to experimental testing in the near future.

## Conflict of interest statement

The authors declare that the research was conducted in the absence of any commercial or financial relationships that could be construed as a potential conflict of interest.

## Footnotes

^1^http://developingmouse.brain-map.org/

^2^http://mouse.brain-map.org

^3^http://mouse.brain-map.org

## Abbreviations

3v: Third ventricle
A/B: Alar-basal boundary
ac: Anterior commissure
ABas: Anterobasal nucleus
ABasM: Median acroterminal ABas
ABasW: Wing (terminal) portion of ABas
Agrp: Agouti-related peptide
AH: hypothalamic nucleus
AHy: Adenohypophysis
Arc: Arcuate nucleus
Arcc: Core portion of the Arc
ArcM: Median acroterminal arcuate nucleus
Arcs: Shell portion of the Arc
ArcW: Wing (terminal) portion of Arc
Avp: Arginin-vasopressin
ATB: Acroterminal border
BFB: Basal-floor boundary
Cartpt: Cocaine- and amphetamine-regulated transcript prepropeptide
ch: Optic chiasm
CPa: Central portion of the peduncular paraventricular area
Crh: Corticotrophin-releasing hormone
DBLH: Dorsobasal lateral hypothalamic area
DM: Dorsomedial hypothalamic nucleus
DM-P: Peduncular DM
DMcP: Core portion of the peduncular DM
DMcT: Core portion of the terminal DM
DMsP: Shell of the peduncular DM
DMsT: Shell portion of the terminal DM
DM-T: Terminal DM
DPa: Dorsal portion of the peduncular paraventricular area
DPM: Dorsal premamillary nucleus
EPD: Dorsal entopeduncular nucleus
EPV: Ventral entopeduncular nucleus
fx: Fornix tract
Gal: Galanin
Ghrh: Growth hormone-releasing hormone
HDB: Hypothalamo-diencephalic boundary
Hcrt: Hypocretin/orexin
IHB: Intrahypothalamic boundary
LH: Lateral hypothalamus
LM: Lateral mamillary nucleus
LPa: Lateral paraventricular nucleus
M: Mamillary region
MCLH: Magnocellular lateral hypothalamic nucleus
ME: Median eminence
mtg: Mamillotegmental tract
NHy: Neurohypophysis
Npy: Neuropeptide Y
opt: Optic tract
os: Optic stalk
Oxt: Oxytocin
Pa: Paraventricular hypothalamic complex
PBas: Posterobasal area and nucleus
Pdyn: Prodynorphin
ped: Peduncle (lateral and medial forebrain bundles)
Penk: Preproenkephalin
PHy: Peduncular hypothalamus
PM: Perimamillary area
Pmch: Pro-melanin-concentrating hormone
PMS: Superficial perimamillary nucleus
POA: Preoptic area
Pomc: Pro-opiomelanocortin α
PPa: Peduncular part of Pa
PRM: Periretromamillary area
PRML: Lateral part of PRM
PRMM: Medial part of PRM
PRtLH: Prereticular lateral hypothalamus
PSPa: Peduncular part of SPa
PSPa lim: Liminal subarea of PSPa
PTh: Prethalamus
PThE: Prethalamic eminence
RM: Retromamillary region
RPa: Rostral paraventricular nucleus
Rt: Reticular nucleus of prethalamus
RTu: Retrotuberal area
RTuD: Dorsal retrotuberal domain
RTuD sub: Subliminal subarea of RTuD
RTuI: Intermediate retrotuberal domain
RTuV: Ventral retrotuberal domain
SCH: Suprachiasmatic nucleus
SCHs: Shell of SCH
sm: Stria medullaris
SPa: Subparaventricular area
SPall: Subpallium
Sst: Somatostatin
STh: Subthalamic nucleus
Th: Tyrosine hydroxylase
Thal: Thalamus
THy: Terminal hypothalamus
TPa: Terminal part of Pa
TPaC: Central portion of TPa
TPaD: Dorsal portion of TPa
TPaV: Ventral portion of TPa
Trh: Thyrotrophin-releasing hormone
TSO: Terminal supraoptic nucleus
TSPa: Terminal part of SPa
Tu: Tuberal region
TuD: Dorsal tuberal domain
TuD sub: Subliminal subarea of TuD
TuI: Intermediate tuberal domain
TuSbO: Tuberal suboptic nucleus
TuV: Ventral tuberal domain
VBLH: Ventrobasal lateral hypothalamic area
Vip: Vasoactive intestinal polypeptide
VM: Ventromedial hypothalamic nucleus
VMc: Core portion of the VM
VMs: Shell portion of the VM
VPa: Ventral portion of the peduncular paraventricular area
VPM: Ventral premamillary nucleus
ZI: Prethalamic zona incerta.
